# Supplementary material for: Cost-utility analysis of robotic-assisted radical cystectomy for bladder cancer compared to open radical cystectomy in the United Kingdom
Source: PLoS One. 2022 Sep 29;17(9):e0270368. doi: 10.1371/journal.pone.0270368 (PMC9522012; doi:10.1371/journal.pone.0270368)
Supplement: S1 Dataset — (PDF) [file pone.0270368.s001.pdf]

Inflation		
Year	CPI	CPI Jan 2021
2016	102.90	119.40
2017	106.60	119.50
2018	110.00	119.60
2019	113.50	119.70
2020	116.60	119.80

				Costs
Item	Service Code		Unit cost	Range (£)
**Pre-operative visit (Consultant led, Non-Admitted Face-to-Face Att**	101	**£**	**127**	
Major Robotic, Prostate or Bladder Neck Procedures (Male), LOS 7-10 days		£	7,820	2,743 - 8,000
**RARC**		**£**	**12,450**	
Cystectomy with Urinary Diversion and Reconstruction, with CC Score 3+		£	12,931	2,733 - 16,285
Cystectomy with Urinary Diversion and Reconstruction, with CC Score 0-2		£	9,293	8,014 - 9,996
**AV ORC**		£	11,112	
Outpatient postoperative day 30 (POD30), (Consultant-led, Non-Admit	101	£	105	
Outpatient postoperative day 90 (POD90) visit	101	£	105	
**SUM follow-up**		£	210	
Single Plasma Exchange or Other Intravenous Blood Transfusion, 19	SA44A	£	773	
**SUM 2 TFs**		**£**	**1,545**	
Infections or Other Complications of Procedures, with Single Interventi	WH07C	£	5,035	856 - 6,221
Infections or Other Complications of Procedures, with Single Interventi	WH07D	£	3,131	997 - 3,510
Complex Infectious Diseases with Single Intervention	WJ01B	£	7,266	1,277 - 11,917
Abdominal Pain with Interventions	FD05A	£	2,556	782 - 3,149
Failure to thrive/dehydration/AKI	CG169	£	2,013	1,257 – 5,111
**AV complications w/ readmission**		**£**	**4,000**	
Outpatient urology	101	£	108	
Outpatient infectious disease	350	£	291	
Outpatient general medicine	300	£	167	
**AV complications w/o readmission**		**£**	**200**	
Regular Day or Night Admission (Major Open Bladder Procedures or R	LB10D	£	197	

Follow-up Examination for Other Conditions, with Interventions	WH53A	£	2,822	994 - 5,310
**SUM no complication readmission**		**£**	**3,019**	

Outcome states	Costs	
RARC with no transfusion, no complication, no readmission	£	14,158
RARC with no transfusion, no complication, readmission	£	17,419
RARC with no transfusion, complication, no readmission	£	14,373
RARC with no transfusion, complication, readmission	£	18,479
RARC with transfusion, no complication, no readmission	£	15,827
RARC with transfusion, no complicaiton, readmission	£	18,846
RARC with transfusion, complication, no readmission	£	16,042
RARC with transfusion, complication, readmission	£	20,148
ORC with no transfusion, no complication, no readmission	£	12,368
ORC with no transfusion, no complication, readmission	£	15,629
ORC with no transfusion, complication, no readmission	£	12,583
ORC with no transfusion, complication, readmission	£	16,689
ORC with transfusion, no complication, no readmission	£	14,037
ORC with transfusion, no complicaiton, readmission	£	17,298
ORC with transfusion, complication, no readmission	£	14,253
ORC with transfusion, complication, readmission	£	18,358

Utilities			
**Health states**	**BL utilities**	**Range**	**Source**
RARC with no complications, readmission or transfusion	0.8	0.6-1.0	Estimated based on Satkuna
ORC with no transfusions, complications, readmissions	0.8	0.6-1.0	Kukreja et al., 2018, Sutton
Transfusion	-0.1	-0.05 to -0.3	Kukreja et al., 2018
Short term complication	-0.3	-0.1 to -0.5	Kukreja et al., 2018, Sutton
Readmission	-0.1	-0.005 to -0.3	Kukreja et al., 2018
Death	0		Assumed

Outcome states	Utilities

RARC with no transfusion, no complication, no readmission	0.8
RARC with no transfusion, no complication, readmission	0.7
RARC with no transfusion, complication, no readmission	0.5
RARC with no transfusion, complication, readmission	0.4
RARC with transfusion, no complication, no readmission	0.7
RARC with transfusion, no complicaiton, readmission	0.6
RARC with transfusion, complication, no readmission	0.4
RARC with transfusion, complication, readmission	0.3
ORC with no transfusion, no complication, no readmission	0.8
ORC with no transfusion, no complication, readmission	0.7
ORC with no transfusion, complication, no readmission	0.5
ORC with no transfusion, complication, readmission	0.4
ORC with transfusion, no complication, no readmission	0.7
ORC with transfusion, no complicaiton, readmission	0.6
ORC with transfusion, complication, no readmission	0.4
ORC with transfusion, complication, readmission	0.3

Probabilities	Base-case	Source
RARC with no transfusion, no complication, no readmission	0.96	Kukreja et al., 2018
RARC with no transfusion, no complication, readmission	0.04	Kukreja et al., 2018
RARC with no transfusion, complication, no readmission	0.64	Kukreja et al., 2018
RARC with no transfusion, complication, readmission	0.36	Kukreja et al., 2018
RARC with transfusion, no complication, no readmission	1	Kukreja et al., 2018
RARC with transfusion, no complicaiton, readmission	0	Kukreja et al., 2018
RARC with transfusion, complication, no readmission	0.71	Kukreja et al., 2018
RARC with transfusion, complication, readmission	0.29	Kukreja et al., 2018
ORC with no transfusion, no complication, no readmission	1	Kukreja et al., 2018
ORC with no transfusion, no complication, readmission	0	Kukreja et al., 2018
ORC with no transfusion, complication, no readmission	0.74	Kukreja et al., 2018
ORC with no transfusion, complication, readmission	0.26	Kukreja et al., 2018

ORC with transfusion, no complication, no readmission	0.75	Kukreja et al., 2018
ORC with transfusion, no complicaiton, readmission	0.25	Kukreja et al., 2018
ORC with transfusion, complication, no readmission	0.68	Kukreja et al., 2018
ORC with transfusion, complication, readmission	0.32	Kukreja et al., 2018

Inflation rate
0.1381910
0.1079498
0.0802676
0.0517962
0.0267112

Min	Min (Infl.)	Max	Max (Infl.)	Total costs (Infl)	Sources	
				**£ 137**	NHS Tariffs 2018/19	
£ 2,743		£ 8,000			NHS Tariffs 2018/19	
	£ 9,655.68		£ 17,931.98	**£ 13,794**	Basal et al. 2018	
£ 2,733		£ 16,285			NHS Tariffs 2018/19	
£ 8,014		£ 9,996			NHS Tariffs 2018/19	
£ 5,374	**£ 5,805**	£ 13,141	**£ 14,195**	**£ 12,004**		
					NHS Tariffs 2018/19	
					NHS Tariffs 2018/19	
				**£ 227**		
£ 611		£ 934			NHS Tariffs 2018/19	
£ 1,222	**£ 1,320**	£ 1,868	**£ 2,018**	**£ 1,669**		
£ 856		£ 6,221			NHS Tariffs 2018/19	
£ 997		£ 3,510			NHS Tariffs 2018/19	
£ 1,277		£ 11,917			NHS Tariffs 2018/19	
£ 782		£ 3,149			NHS Tariffs 2018/19	
£ 1,257		£ 5,111			NICE	Altobelli et al, 2017
£ 1,034	**£ 1,117**	£ 5,982	**£ 6,462**	**£ 4,321**		
£ 88		£ 111			NHS Tariffs 2018/19	
£ 233		£ 303			NHS Tariffs 2018/19	Radical Cystectomy follow up
£ 98		£ 185				
£ 140		£ 200		**£ 216**		
£ 197		£ 197			NHS Tariffs 2018/19	

£	994			£	5,310					NHS Tariffs 2018/19
£	1,191	**£**	**1,287**	£	5,507	**£**	**5,949**	**£**	**3,261**	

ısivam et al.

et al. 2018

et al. 2018

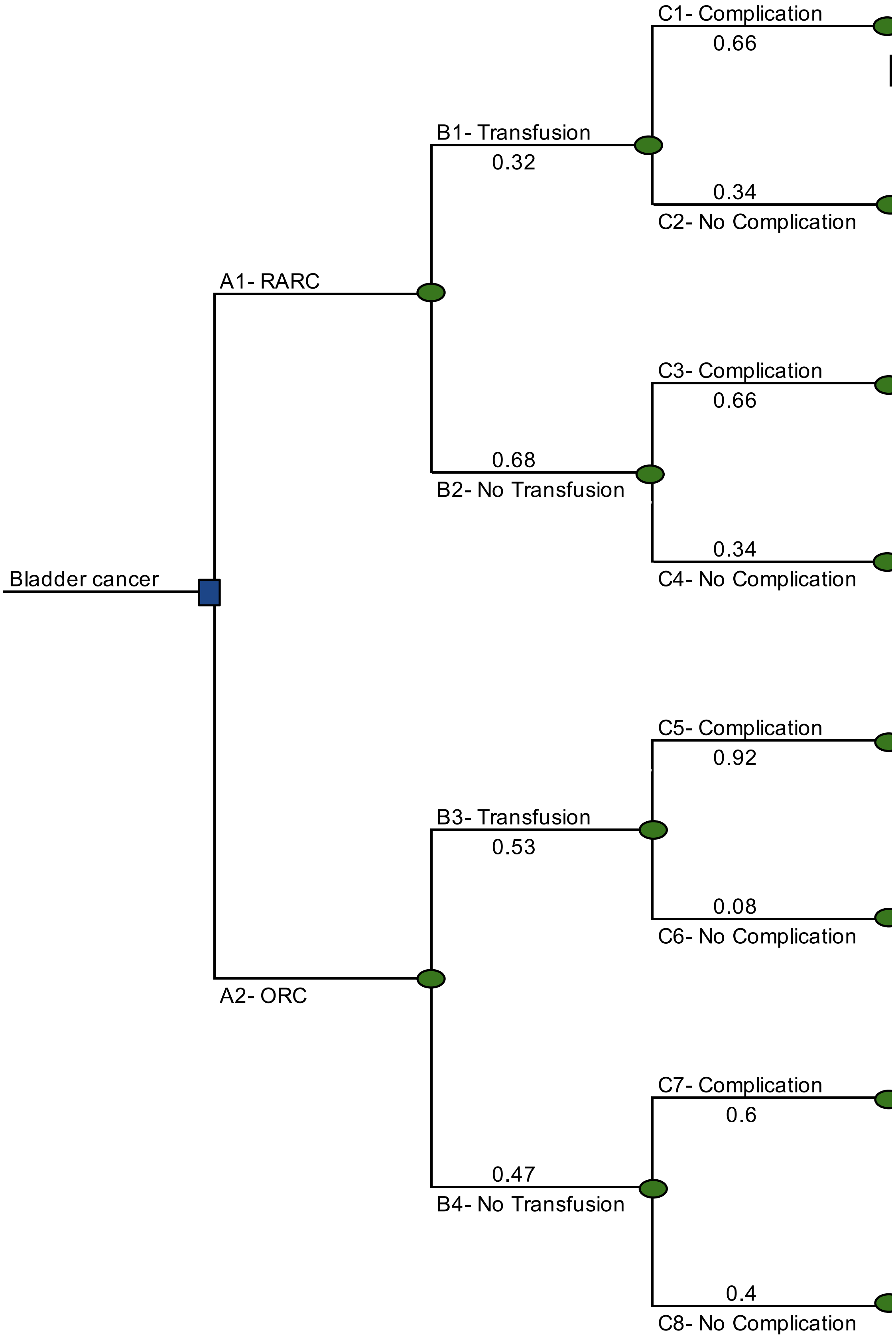

Bladder cancer
A1- RARC
A2- ORC
B1- Transfusion
0.32
0.68
B2- No Transfusion
B3- Transfusion
0.53
0.47
B4- No Transfusion
C1- Complication
0.66
0.34
C2- No Complication
C3- Complication
0.66
0.34
C4- No Complication
C5- Complication
0.92
0.08
C6- No Complication
C7- Complication
0.6
0.4
C8- No Complication

Branch	Probability	Cost	Outcome	Cond Prob	EX COSTS	EX QALYs
D1- Readmission	0.29	£ 20,148	0.3	0.061	£ 1,234	0.018
D2- No Readmission	0.71	£ 16,042	0.4	0.150	£ 2,406	0.060
D3- Readmission	0	£ 18,846	0.6	0.000	-	0.000
D4- No Readmission	1	£ 15,827	0.7	0.109	£ 1,722	0.076
D5- Readmisison	0.36	£ 18,479	0.4	0.162	£ 2,986	0.065
D6- No Readmission	0.64	£ 14,373	0.5	0.287	£ 4,128	0.144
D7- Readmission	0.04	£ 17,419	0.7	0.009	£ 161	0.006
D8- No Readmission	0.96	£ 14,158	0.8	0.222	£ 3,142	0.178
					**£ 15,779**	**0.547**
D9- Readmission	0.32	£ 18,358	0.3	0.156	£ 2,864	0.047
D10- No Readmission	0.68	£ 14,253	0.4	0.332	£ 4,726	0.133
D11- Readmission	0.25	£ 17,298	0.6	0.011	£ 183	0.006
D12- No Readmission	0.75	£ 14,037	0.7	0.032	£ 446	0.022
D13- Readmission	0.26	£ 16,689	0.4	0.073	£ 1,223.66	0.029
D14- No Readmission	0.74	£ 12,583	0.5	0.209	£ 2,625.92	0.104
D15- Readmission	0	£ 15,629	0.7	0.000	£ -	0.000
D16- No Readmission	1	£ 12,368	0.8	0.188	£ 2,325.18	0.150
					**£ 14,394.66**	**0.492**

	Intervention	Control	Difference
Expected cost	£15,779	£14,395	£1,385
Expected outcome QALYs	0.547	0.492	0.055
**ICER**			**25,325.96**
**NMB (£20,000 per QALY)**	-4,843	-4,552	-291
**NMB (£30,000 per QALY)**	625	369	256
NHB (£20,000 per QALY)	-0.242	-0.228	
NHB (£30,000 per QALY)	0.021	0.012	

Threshold £ 20.000/QALY		ICER	
**Δ costs**	**Δ effects**	**Δ costs**	**Δ effects**
0	0	0	0
£0	1,385	£0	1,385

Δ Costs	old £ 20.000/QA	ICER
0.000	0	
0.069	1,385	
0.000		0
0.055		1,385

Ceiling ratio	NMB	NHB
£0	-15,779	#DIV/0!
£5,000	-13,045	-3.156
£10,000	-10,311	-1.578
£15,000	-7,577	-1.052
£20,000	-4,843	-0.789
£25,000	-2,109	-0.631
£30,000	625	-0.526
£35,000	3,359	-0.451

£40,000	6,093	-0.394
£45,000	8,827	-0.351
£50,000	11,560	-0.316
£55,000	14,294	-0.287
£60,000	17,028	-0.263
