# Supplementary material for: Cost-utility analysis of robotic-assisted radical cystectomy for bladder cancer compared to open radical cystectomy in the United Kingdom
Source: PLoS One. 2022 Sep 29;17(9):e0270368. doi: 10.1371/journal.pone.0270368 (PMC9522012; doi:10.1371/journal.pone.0270368)
Supplement: Attachment — Submitted filename: Response to Reviewers.docx [file pone.0270368.s002.docx]

**Response to reviewers**

**Reviewer Comment 1:** *The ICER for robotic versus open cystectomy was close, although did not meet the threshold of 20,000 pound/QALY. Given how close it is and the finding that RARC did improve QALY, I would suggest also exploring what modifications would have to exist to reach this level.*

**Response:** We thank you for this suggestion. Based on the input parameters (probabilities, benefits and costs), the sensitivity analysis and tornado diagram showed that the ICER was mainly influenced by the cost of RARC (see figure 6). On this basis, the linear regression model of the ICER for RARC showed that if the cost of the intervention was reduced to £13.497, the threshold of £20,000/QALY would be reached (see figure 5). We added one sentence in the discussion of the results to highlight the modification of costs to make the ICER reach this level.

**Reviewer Comment 2:** *Were choice and approach of urinary diversion accounted for? Many of the RARC studies included an open incision for the urinary diversion. What are the implications for an intracorporeal neobladder or ileal conduit?*

**Response:** Thank you for raising this additional point. As for the parameters used to model the ICER, the probabilities and utilities (based mainly on Kukreja et al. 2018) refer to radical cystectomies (RARC and ORC) containing an ileal conduit for urinary diversion (see Kukreja et al. 2018). We included an explanatory sentence in the methods section to reflect on this. We could not find data on probabilities and utilities that refer to the implications of an intracorporeal neobladder.
